# Which factors affect the implementation of geriatric recommendations by primary care physicians?

**DOI:** 10.1186/s13584-017-0134-7

**Published:** 2017-04-25

**Authors:** Yan Press, Boris Punchik, Ella Kagan, Alex Barzak, Tamar Freud

**Affiliations:** 1 Department of Family Medicine, Siaal Research Center for Family Medicine and Primary Care, Faculty of Health Sciences, Ben-Gurion University of the Negev, Beer-Sheva, Israel; 2 Yasski Clinic, Comprehensive Geriatric Assessment Unit, Clalit Health Services, Beer-Sheva, Israel; 3 Unit for Community Geriatrics, Division of Health in the Community, Ben-Gurion University of the Negev, Beer-Sheva, Israel

**Keywords:** Outpatient, Assessment, Adherence

## Abstract

**Background:**

The overall implementation rate for outpatient comprehensive geriatric assessment (OCGAU) recommendations ranges from 48.6 to 71%.

The purpose of the study was to identify factors that reduce the implementation rate of geriatric recommendations.

**Methods:**

The medical records of patients who were assessed in the comprehensive geriatric assessment unit over an 8 year study period were surveyed. Data collected included patient's characteristics (socio-demographic, functional, cognitive, and affective condition, co-morbidity), number of recommendations, the identity of the geriatrician, and data related to the primary physician (age, sex, seniority, number of patients referred for geriatric assessment).

**Results:**

Three thousand four hundred thirty-four recommendations were made for 488 patients (mean age 83.6 ± 0.6 years) of which 1,634 (47.6%) were implemented by their primary physician. In univariate analyses patients with an implementation rate < 25%, compared to patients with implementation rate ≥75%, had a higher Charlson Comorbidity Index Total Score (CCITS) (2.5 ± 1.9 vs. 1.8 ± 1.7, *P* < 0.05), a lower Barthel Index (82.8 ± 16.2 vs. 87.0 ± 15.3, *P* < 0.05), and a lower Instrumental Activity of Daily Living score (7.2 ± 3.5 vs. 8.2 ± 3.7, *P* < 0.05). There were no differences between these groups in other patient characteristics or the number of recommendations made during the assessment. Similarly, there were no differences in the identity of the geriatrician or the primary physician's characteristics. In the multivariate analysis only higher CCITS was associated with a lower rate of recommendation implementation by primary physicians.

**Conclusions:**

There is a need to increase the implementation rate by primary physicians by increasing and strengthening the link with them and by further training in the field of geriatrics medicine.

**Trial registration:**

The Helsinki committee of the Meir Medical Center approved the study (Approval #024/2015 [k]).

## Background

The effectiveness of outpatient comprehensive geriatric assessment (OCGA) has been studied frequently, but without clear-cut conclusions [1–14].

The OCGA process includes the following steps: 1) selecting appropriate patients for assessment (targeting), 2) comprehensive assessment, and 3) provision and implementation of recommendations. Each step in the process contributes to its overall effectiveness [15]. In some OCGA models the implementation of recommendations is the responsibility of the primary physician, so a low implementation rate could explain, at least in part, the lack of effectiveness of interventions [5, 7, 15]. Thus, efforts have been made to improve the implementation rate of primary physicians [9].

In the present study we investigated factors associated with the implementation by primary physicians of recommendations reached during OCGA.

## Methods

### Study aims

Identifying factors reducing the implementation rates of geriatric recommendations.

### The unit staff

The OCGA unit (OCGAU) of the southern region of the Clalit Health Services was established in Beer-Sheva in 2004. The unit is active about 15 h per week and consists of a permanent core of four geriatrics specialists, a nurse, a social worker, and a secretary. At various times other professionals also participate in the unit’s activity including an occupational therapist, a physical therapist, dieticians, a clinical pharmacologist, and volunteers.

### Patients

The Helsinki committee of the Meir Medical Center approved the study (Approval #024/2015 [k]).

The unit assesses patients 65 years of age and above who were referred by their primary physicians. The patients are referred by the primary physician because of complex medical issues or the presence of functional, cognitive, mental, or social problems.

### The OCGAU work process and data recording

The patient’s referral form, together with the computerized medical record, are sent to the unit by internal email. The policy of the unit is to invite the patient together with the caregiver and to emphasize the importance of the caregiver’s attendance so that, except in the minority of cases in which the patients has no caregiver, the caregiver is always present at the assessment in the unit.

The OCGAU staff collects information on the patient's socio-demographic details, habits such as smoking, alcohol consumption and physical activity, falls, and sleeping habits and also completes and obtains supplemental information on vaccinations, and allergies to drugs and other substances. Patient undergo measurement of height, weight and blood pressure, and a functional assessment using the Older Americans Resources and Service Instrumental Activity of Daily Living (IADL-OARS) [16] and the Barthel Index (BI) [17]. A cognitive assessment is conducted using the Mini-Mental Examination (MMSE) [18], the clock drawing and, if needed, the Montreal Cognitive Assessment (MoCA) [19], and a neuro-cognitive assessment using the Mindstreams program [20]. The staff also conducts an affective assessment. Over the years we have transitioned from the 15-item Geriatric Depression Scale (GDS-15) [21] to the Patient Health Questionnaire (PHQ-9) [22]. The staff conducts an assessment of support systems, living conditions, sources of income, and principal caregiver distress. At the end of the process the geriatrician completes the history with a focus on the complaints of the patient and the primary caregiver, a medication assessment (over part of the study period this was done by a clinical pharmacologist), and chronic comorbidity by calculating the Charlson: comorbidity index (CCI) [23]. The geriatrician also conducts a physical examination and a mobility assessment.

A comprehensive report is written at the end of the assessment. The core of the summary is presented systematically to the patient and the caregiver during a dedicated 20–25 min session with an emphasis on the need for implementation of the recommendations. At the end of the session the patient receives a hard copy of the most important recommendations. The patient and the caregiver are encouraged to go over the summary again at home. The patient is asked to make a long appointment with the primary physician to give the doctor enough time to read the summary and discuss it with the patient.

The full summary is sent by email to the primary physician. Other than in unusual cases the geriatrician does not have a telephone conversation with the primary physician. The staff of the geriatric assessment unit aspires to base its recommendations on evidence-based medicine. In order to guarantee quality assurance the initial recommendations provided by the geriatrician who did the assessment are discussed on a regular basis at the weekly staff meeting that is attended by all the members of the staff.

### Recording of recommendation implementation by the primary physician

In the present study we evaluated only implementation of recommendations by the primary physician, not the patient. For example, if according to our recommendation the primary physician referred the patient to a physical therapist that would be considered implementation of a recommendation. We did not check the actual implementation of the recommendation, i.e., whether the patient actually went to the physical therapist or how many treatment sessions they attended.

The implementation rates were recorded for the following types of recommendations: change in dose or discontinuation of drugs, referral for laboratory or imaging tests, referral to additional consultant physicians, referral for physical therapy, occupational therapy, social services (defined as non-MD referral) and completion of vaccination requirements.

The rate of recommendation implementation was calculated as percentages in each category. For example, if there was a recommendation to add two drugs and to discontinue three drugs and the primary physician added one drug and discontinued one drug the rate of implementation for adding drugs would be 50% and for discontinuation of drugs would be 33.3%, with an overall implementation rate for pharmacological recommendations of 40% (2 of 5 drugs with any recommended change). A final total rate of recommendation implementation was then calculated.

Information on the implementation of recommendations was collected from the patient’s medical record. In our previous study [24], conducted in clinics in Beer-Sheva, we found that 94% of the recommendations implemented by the primary physician were implemented over the first three months following the geriatric assessment (unpublished data). Based on this finding, we only surveyed the medical records for this period of time in the present study. In this retrospective cross-sectional study we included all patients who underwent a comprehensive geriatric assessment in the unit setting between January 2005 and December 2013. We did not include patients who changed clinics, left the region, or whose computerized record could not be accessed for any reason.

### Statistical analyses

The patients were categorized into four groups based on the degree of overall implantation of recommendations as follows: 1Q = 0–24%, 2Q = 25–49%, 3Q = 50–74% and 4Q = 75% and above. Two comparisons were conducted. The first was among all four groups and the second between groups 1Q (minimal implementation) and 4Q (maximal implementation).

Categorical variables are described as frequencies and percentiles. Continuous variables, such as age, are described as mean ± standard deviation (SD). Differences in categorical variables were tested using the Chi-square or Fisher exact test in accordance with the size of the cells. Differences in continuous variables were tested by one-way ANOVA. A regression model was constructed to predict patients for whom more than 75% of the recommendations were implemented. The model included age, sex, and variables that were found to have a statistically significant difference in comparisons between groups 1Q and 4Q. In all statistical tests *P* < 0.05 was considered to be statistically significant.

## Results

Over the study period 628 patients underwent an assessment at the OCGAU. Data on the implementation of geriatric recommendations by primary physicians was found for 502 of these patients. The reason for exclusion of 126 patients in the sample was that the data on the implementation of recommendations was collected from the computerized medical records of the patients. The Clalit Healthcare Services does not have a uniform medical record. Each patient’s medical record is stored on the server of the clinic where they are registered. In order to collect the data the investigators had to go to the clinic and check the medical record. For this reason, the records of patients who left the area, or who transferred to another clinic or another healthcare service were not available to investigators and were not included in the study.

Since no recommendations were made by the unit staff for 14 of the 502 patients the final sample included 488 patients or 77.7% of the 628 patients.

No statistically significant differences were found in age, sex, functional status, comorbidity, or the number of medications between the 488 patients included in the study and the 140 who were not included.

The mean age of the 488 patients was 83.6 ± 0.6 years and 34.4% were men. Of the 764 recommendations to increase the dose of an existing medication or to begin a new one, 433 (56.7%) were implemented. Of the 650 recommendations to decrease the dose of an existing medication or to discontinue one, 316 (48.6%) were implemented. In total the implementation rate for “pharmacological” recommendations was 53.0% (749 of 1414). Table 1 presents the 10 most common drugs for which there was a recommendation to either add the drug or increase the dose (62% of the recommendations of this type) and the 10 most common drugs for which there was a recommendation to either discontinue the drug or reduce the dose (78% of the recommendations of this nature).

**Table 1:** Ten more frequent recommendation to add/increase dose of drug or stope/decrease dose of drug, and rates of implementations

Type of recommendations	N recommendations	N of implemented recommendations	Implementation rate
*Add/increase dose of drug*			
Calcium and vitamin D supplementation	174	100	57.5
Antidepressants	169	97	57.4
Cholinesterase inhibitors/ Memantine	72	54	75.0
Non-opioid analgetics	56	30	53.6
Antiresorptive drugs	33	17	51.5
ACE- inhibitors	23	10	43.5
Statins	22	12	54.5
Beta- blockers	18	6	33.3
Neuroleptics	15	9	60.0
Diuretics	14	2	14.3
*Stop/decrease dose of drug*			
Benzodiazepines/ Z-drugs	91	39	42.9
Multivitamins	58	25	43.1
Diuretics	53	30	56.6
Calcium channel blockers	44	15	34.1
Statins	33	19	57.6
Sulfonylurea	27	12	44.4
Anticholinergic agents	26	12	46.2
Nitrates	23	8	34.8
ACE-inhibitors	23	10	43.5
Propoxyphene	23	18	78.3

Two hundred seventy eight of the 466 recommendations to conduct a laboratory test were implemented (59.7%). The implementation rate for imaging or other diagnostic tests (other than laboratory tests) was 48.4% (180 of 372), the implementation rate for non-MD referrals was 41.2% (175 of 425), for referrals to a consultant doctor 35.6% (110 of 309), and for completion of vaccination recommendations 31.7% (142 of 448). In all, 3,434 different recommendations were made for the 488 study participants (mean = 7.0 ± 3.6 recommendations per patient, range 1–19) and primary physicians implemented 1,634 recommendations (47.6%).

### Recommendation implementation – patient characteristics

The socio-demographic and health-related characteristics of the patients in the entire study population and in the sub-groups are presented in Table 2. There were no significant differences between the groups in age, sex or family status. There were also no differences in functional status (OARS-IADL, BI) among the four groups, but in a comparison of groups 1Q and 4Q the patients in 1Q had a significantly lower level of basic functioning as measured by BI (82.8% ± 16.2% vs. 87.0% ± 15.3%, *P* = 0.027) and in instrumental functioning as measured by OARS-IADL (7.2% ± 3.5% vs. 8.2% ± 3.7%, *P* = 0.03).

**Table 2:** Socio-demographic and health-related characteristics of patients in the entire study group and in the sub-groups categorized by recommendation implementation

		Q1- Implementation 0–24% (*N* = 124)		Q2- Implementation 25–49% (*N* = 103)		Q3- Implementation 50-50–74% (*N* = 143)		Q4- Implementation 75–100% (*N* = 118)		P*	P**
		*N*	%	*N*	%	*N*	%	*N*	%		
Gender	Male	39	31.5	37	35.9	54	37.8	38	32.2	0.67	0.98
	Female	85	68.5	66	64.1	89	62.2	80	67.8		
		124		103		143		118			
Age (years)	Mean ± SD	83.7 ± 6.5		83.8 ± 6.0		83.6 ± 5.5		83.5 ± 5.9		0.972	0.775
	Range	67–100		70–103		69–97		67–96			
		124		103		143		118			
Family Status	Married	47	42.7	44	49.4	55	48.2	47	51.6	0.62	0.263
	Other	63	57.3	45	50.6	59	51.8	44	48.4		
		110	(mis = 14)	89	(mis = 14)	114	(mis = 29)	91	(mis = 27)		
Education (years)	Mean ± SD	8.8 ± 5.2		9.8 ± 4.7		9.5 ± 5.2		9.3 ± 4.9		0.489	0.477
	Range	0–20		0–20		0–20		0–20			
		114	(mis = 10)	93	(mis = 10)	131	(mis = 12)	96	(mis = 22)		
Barthel Index (0–100)	Mean ± SD	82.8 ± 16.2		84.1 ± 16.9		84.7 ± 15.8		87.0 ± 15.3		0.149	0.027
	Range	35–100		25–100		30–100		35–100			
		115	(mis = 9)	96	(mis = 7)	132	(mis = 11)	115	(mis = 3)		
OARS- IADL^a^ (0–14)	Mean ± SD	7.2 ± 3.5		7.4 ± 3.7		7.4 ± 3.2		8.2 ± 3.7		0.126	0.03
	Range	0–14		0–14		0–14		14-ינו			
		116	(mis = 8)	96	(mis = 7)	135	(mis = 8)	115	(mis = 3)		
MMSE^b^ (0–30)	Mean ± SD	21.33 ± 5.88		22.42 ± 5.76		21.58 ± 5.41		23.02 ± 4.71		0.106	0.052
	Range	6–30		2–30		6–30		8–30			
		109	(mis = 15)	96	(mis = 7)	137	(mis = 6)	113	(mis = 5)		
CDT^c^ (0–10)	Mean ± SD	6.4 ± 3.2		6.6 ± 2.9		6.2 ± 2.9		6.7 ± 2.9		0.56	0.51
	Range	0–10		0–10		0–10		0–10			
		111	(mis = 13)	98	(mis = 5)	137	(mis = 6)	115	(mis = 3)		
High DS^d^	Yes	81	83.5	68	85.0	96	79.3	76	79.2	0.65	0.55
	No	16	16.5	12	15.0	25	20.7	20	20.8		
		97	(mis = 27)	80	(mis = 23)	121	(mis = 22)	96	(mis = 22)		
CCITS^e^	Mean ± SD	2.5 ± 1.9		2.4 ± 2.0		2.2 ± 1.7		1.8 ± 1.7		0.029	0.0044
	Range	0–8		0–11		0–8		0–8			
		105	(mis = 19)	96	(mis = 7)	117	(mis = 26)	92	(mis = 26)		
Number of medications per month	Mean ± SD	7.87 ± 4.43		8.35 ± 4.28		8.13 ± 4.32		8.19 ± 4.41		0.768	0.434
	Range	1.0–23.7		1.0–23.7		1.3–26.0		0.7–29.7			
		115	(mis = 9)	94	(mis = 9)	132	(mis = 11)	105	(mis = 13)		

P* among all Q groups, P** between Q1/Q4

^a^OARS-IADL- the Older Americans Resources and Service Instrumental Activity of Daily Living; ^b^MMSE- Mini-mental State Examination; ^c^CDT-Clock drawing test; ^d^High DS- High depression score: a composite score comprised of patients with a GDS-15 score ≥ 5, or a PHQ-9 score ≥10; ^e^CCITS- the Charlson Comorbidity Index- Total Score

There were no significant differences in cognitive state, measured by MMSE, between the four groups and between 1Q/4Q, although a trend was seen in which MMSE was higher in 4Q compared to 1Q (23.02 ± 4.71 vs. 21.33 ± 5.88, *P* = 0.052).

Because over the study years we transitioned from the GDS-15 instrument to the PHQ-9, the two tests were combined and a High Depression Score, defined as GDS-15 ≥ 5 or a PHQ-9 score ≥ 10, was calculated. There were no significant differences among the four groups or in the 1Q/4Q comparison for this combined score.

A significant difference was seen in comorbidity, measured by the CCITS, in the comparisons of both the four groups and between 1Q and 4Q (2.5 ± 1.9 vs. 1.8 ± 1.7, *P* = 0.0044).

### Characteristics of the CGA, per se

Table 3 shows the distribution of types of recommendations per implementation group. There were no significant differences among the four groups or in the 1Q/4Q comparisons in any of the recommendation types, except for completion of vaccinations. There were no significant differences in terms of the geriatrician (data not shown) or the number of recommendations given (Q1 = 7.2 ± 3.5, Q2 = 7.1 ± 3.3, Q3 = 7.3 ± 3.7, Q4 = 6.5 ± 3.4, *P* = 0.27, P [Q1/Q4 = 0.11]).

**Table 3:** Distribution of types of recommendation, by implementation group

	Q1- Implementation 0–24% (*N* = 124)			Q2- Implementation 25–49% (*N* = 103)			Q3- Implementation 50–74% (*N* = 143)			Q4- Implementation 75–100% (*N* = 118)			All (*N* = 488)			P*	*P* **
	Mean ± SD	Range	*N*	Mean ± SD	Range	*N*	Mean ± SD	Range	*N*	Mean ± SD	Range	*N*	Mean ± SD	Range	*N*		
Add/increase dose of drug	2.0 ± 1.0	1–5	97	1.9 ± 1.0	1–5	87	2.1 ± 1.2	1–5	111	1.9 ± 1.1	1–5	94	2.0 ± 1.1	1–5	389	0.65	0.44
Stop/decrease dose of drug	2.3 ± 1.5	1–7	74	2.1 ± 1.3	1–7	58	2.3 ± 1.5	1–7	90	2.0 ± 1.4	1–6	68	2.2 ± 1.5	1–7	290	0.86	0.98
All pharmacologic recommendations	3.3 ± 1.8	1–9	110	2.9 ± 1.75	1–9	96	3.2 ± 2.1	1–10	137	3.2 ± 1.9	1–9	103	3.2 ± 1.9	1–10	446	0.63	0.76
Laboratory tests	2.1 ± 1.2	1–6	52	1.7 ± 1.1	1–7	53	2.3 ± 1.3	1–5	62	2.0 ± 1.2	1–7	63	2.0 ± 1.2	1–7	230	0.08	0.78
Imaging and other diagnostic tests	1.4 ± 0.6	1–4	73	1.4 ± 0.7	1–4	61	1.5 ± 0.8	1–5	71	1.6 ± 0.7	1–3	50	1.5 ± 0.7	1–5	255	0.61	0.15
“MD” referral	1.5 ± 0.7	1–1	56	1.5 ± 0.6	1–3	63	1.6 ± 0.9	1–4	63	1.4 ± 0.7	1–3	45	1.5 ± 0.7	1–4	207	0.44	0.52
“Non-MD” referral	1.4 ± 0.6	1–1	90	1.4 ± 0.5	1–2	64	1.3 ± 0.5	1–3	90	1.3 ± 0.5	1–3	71	1.4 ± 0.5	1–4	315	0.53	0.42
Immunization	1.9 ± 0.8	1–3	62	1.9 ± 0.8	1–3	60	1.8 ± 0.8	1–3	77	1.4 ± 0.5	1–3	53	1.8 ± 0.8	1–3	252	0.019	0.002
Total	7.2 ± 3.5	1–17	124	7.1 ± 3.3	3–18	103	7.3 ± 3.7	2–18	143	6.5 ± 3.6	1–15	118	7.0 ± 3.6	1–18	488	0.27	0.11

P* among all Q groups, P** between Q1/Q4

### Recommendation implementation – characteristics of the primary physician

Ninety eight doctors referred their patients for assessment at the unit, a mean of 4.9 ± 4.7 patients per doctor (range 1–29). All the patients were categorized into four groups based on the number of patients who were referred to the unit by their doctor during the study period: group 1 (113 patients of 57 doctors who referred 1–4 patients to the unit), group 2 (135 patients of 18 doctors who referred 5–7 patients), group 3 (108 patients of 16 doctors who referred 8–11 patients), and group 4 (8 doctors who referred 12 or more patients to the unit). We found no difference in the rate of implementation of geriatric recommendations by primary physicians when groups 1 and 4 were compared (55.1 ± 30.5 vs. 54.5 ± 30.1, respectively, *P* = 0.87).

We evaluated associations between primary physicians’ age, sex, seniority, and specialization and recommendation implementation. As can be seen in Table 4 no differences were found in these variables among the four groups.

**Table 4:** Comparison of primary physicians by implementation group

Physician characteristics		Q1- Implementation 0–24% (*N* = 124)		Q2- Implementation 25–49% (*N* = 103)		Q3- Implementation 50–74% (*N* = 143)		Q4- Implementation 75–100% (*N* = 118)			All (*N* = 488)	P*	P**
		*N*	%	*N*	%	*N*	%	*N*	%	*N*	%		
Gender	Male	35	28.2	20	19.4	30	21.0	29	24.6	114	23.4	0.38	0.56
	Female	89	71.8	83	80.6	113	79.0	89	75.4	374	76.6		
		124		103		143		118		488			
Experience (years)	Mean ± SD	14.2 ± 7.0		14.1 ± 6.1		14.3 ± 6.6		14.5 ± 6.6		14.3 ± 6.6		0.81	0.69
	Range	2–39		2–35		2–36		3–37		2–39			
		123	(mis = 1)	100	(mis = 3)	138	(mis = 5)	111	(mis = 7)	472	(mis = 16)		
Specialty	Family Medicine	91	73.4	72	69.9	101	70.6	79	66.9	343	70.3	0.52	0.3
	General Medicine	11	8.9	18	17.5	21	14.7	18	15.3	68	13.9		
	No specialization	22	17.7	13	12.6	21	14.7	21	17.8	77	15.8		
		124		103		143		118		488			

P* among all Q groups, P**between Q1/Q4

### General model

A logistic model was developed to predict patients with an implementation rate ≥ 75%. It included age, sex, and other characteristics that were found to significantly differ between the groups with the minimal and maximal implementation rates. Based on this approach CCITS, MMSE, OARS-IADL and BI should have been entered into the model. In order to avoid overloading the model and because there was a statistically significant correlation between the two functional indices (*r* = 0.725, *P* < 0.001) we decided to enter only BI as an index of function. The final model included age, sex, BI, MMSE, and CCITS. Only CCITS was found to be associated indirectly with a higher rate of implementation of geriatric recommendations by the primary physician (OR = 0.82, CI 95%: 0.706-0.968, *P* = 0.018).

## Discussion

In the present study we found that the rate of implementation of OCGAU recommendations was 47.6%, with “pharmacological” recommendations and recommendations for laboratory tests implemented most (53% and 59.7%, respectively) and recommendations to refer to another consultant (35.6%) or to complete vaccinations (31.7%) the least implemented.

Table 1 shows that some of the pharmacological recommendations are important, but difficult to implement, for example discontinuation of benzodiazepines with an implementation rate of 42.9%. Some of the recommendations had a high implementation rate, for example discontinuation of propoxyphene with a rate of 78.3%. Primary care physicians may have perceived some of the recommendations as less important so the rate of implementation for them was lower, for example discontinuation of multivitamins with an implementation rate of 43.1%. However, there apparently were other reasons for the relatively low implementation rate for some of the other recommendations despite the clear rationale behind these recommendations, for example less than 60% of the patients with osteoporosis got a recommendation to begin treatment with calcium, vitamin D and anti-resorptive therapy. Unfortunately, in a retrospective study it is impossible to evaluate the motivation of primary care physicians to implement specific recommendations or not.

Primary care physicians know their patients, their patients’ families and the surroundings better than any consulting doctor. In light of this acquaintance with patients it is possible that in some cases not implementing a recommendation may bring more benefit to the patient than implementing it. Thus, the implementation rate of geriatric recommendations by primary care physicians does not necessarily have to be 100%, making it difficult to determine an optimal implementation rate for geriatric recommendations. However, if the assumption that there is a direct association between implementation of recommendations and the effectiveness of the comprehensive geriatric assessment is correct, it is reasonable to aspire to a situation in which the majority of recommendations are implemented.

The implementation of OCGAU recommendations has been surveyed extensively because there is a significant association between the success of interventions and the implementation of recommendations by primary physicians, with an overall implementation rate ranging from 48.6% to 71% [9, 15, 25–27]. Previous studies also showed that recommendations to change drug therapy [26–28] or for further tests [26] are the most implemented while recommendations relating to preventive medicine are the least implemented [26]. Because of significant differences between different types of OCGAU settings in terms of population, staff, geographic region, clinic working hours, the place of geriatric medicine in the overall healthcare system, *i.a*. and the great difference in study methodology among the various studies, it is very difficult to compare the present results with those of other studies. Furthermore, the goal of the present study was different than most others, i.e., to identify characteristics of the patient population, the unit staff and primary physicians that were associated with a higher rate of implementation of geriatric recommendations.

### The process of patient referral, evaluation of the suitability of the patient to the setting and the comprehensive geriatric assessment

Patients can come to the OCGAU only if they are referred by their primary physician, so the doctor was involved in or even initiated the process of patient referral. All the patients that came to the unit underwent an identical process of selection, so the selection process itself could not have been the cause of the variability in recommendation implementation.

Although the unit staff changed during the course of the study (physical therapist, occupational therapist, and clinical pharmacologist), the patients underwent a standard assessment that did not change over the study years. Thus, it is not likely that the differences found in the implementation of recommendations were associated with the actual assessment process in the unit. As already mentioned, we did not find any difference in the implementation of recommendations by different geriatricians. A previous study of geriatric consultation in Beer-Sheva clinics also did not find that the identity of the geriatric consultant affected the implementation of recommendations by primary physicians [29].

### The effect of patient characteristics on the implementation of recommendations

In the present study, as in previous studies, there was no association between patient age [30] or sex [15, 30] and the rate of implementation of recommendations.

In the unilateral analysis we found an association between a low rate of implementation of recommendations and functional state, cognitive state, and burden of comorbidity, but in the logistic regression model only burden of comorbidity maintained a statistically significant association. It is not clear why patients who are in greater need of intervention have a lower rate of recommendation implementation. According to Winograd and Stearns [31] geriatric problems are usually chronic and multidimensional, and the implementation of geriatric recommendations can take a lot of time and resources, so the primary physician may not consider the implementation of recommendations to be cost-effective. This attitude on the part of the primary physician may be even stronger when it comes to elderly patients.

The implementation of recommendations was evaluated over the first three months after the recommendations were given. Only five of the 488 patients died during the course of this period. The mean number of recommendations per patient for these patients was 7 (range 3–10). In four of the five patients none of the recommendations was implemented. Sixteen of the 488 patients died within 4–12 months of the geriatric assessment. The implementation rate for recommendations in these patients was 49.6%. Thus, even if the family doctor had a sense that the life expectancy of these patients would be short it did not effect the implementation of recommendations.

One assumption is that limiting the number of recommendations to a minimum increases the rate of implementation [27, 32]. However, in a follow-up study to the initial study by Sears and Charlson [32] in which they found an inverse association between the number of recommendation given and their implementation, the investigators tried to limit the number of recommendations to five, but did not find a subsequent change in the rate of implementation of recommendations [32]. Using a univariate model Reuben *i.a*. found an association between the number of recommendations and their implementation, but this association disappeared in the multivariate model [15]. Bogardus *i.a*. also did not find and association between the number of recommendations and their implementation by the primary physician [33]. In the present study we did not find such an association as well.

### Characteristics of the primary physician

In the present study we did not find any association between the age, sex, or seniority of the primary physician and the implementation of geriatric recommendations.

In a previous study we evaluated possible associations between these variables among primary physicians, some of whom also participated in the present study, and did not find any association between the characteristics of the primary physician and the implementation of geriatric recommendations [29]. The association between the primary physician’s age and the implementation of geriatric recommendation has been evaluated in the past with contradictory results [30, 34]. Similarly contradictory results were found in studies of a possible association of the doctor’s sex [30, 35]. Bula *i.a*. found that doctors with less seniority had a higher rate of implementation of geriatric recommendation [36], but this association was not found the in the present study.

Previous studies have shown that primary physicians are more likely to implement recommendations that they perceive to be easier to implement [27, 34, 37] and recommendations that they perceive to be more beneficial for their patients [36]. It is reasonable to assume that doctors who felt in the past that geriatric recommendations were either too difficult or not beneficial for their patients would be less likely to implement these recommendation and less likely to refer their patients to the unit in the future. In contrast, doctors who refer their patients to the unit frequently are more likely to implement the recommendations. To test the assumption that doctors who refer patients more frequently also implement recommendation more, we performed an analysis of the number of patients referred to the unit by each doctor. No association was found between the implementation of geriatric recommendation and the number of patients referred by the doctor for assessment. This finding strengthens the impression that “implementation habits” are not associated with doctor characteristics. Otherwise why would doctors refer patients over and over again for geriatric assessment if they do not believe in the necessity of the assessment itself?

## Conclusions

The results of this study indicate a need for an intervention to increase the implementation rate by primary physicians. Sternberg and Bentur [38] evaluated the contribution of the CGA for 200 primary care physicians who sent at least six of their patients for a geriatric assessment. They found that only 36% of the respondents were very satisfied about the diagnosis and the drug-related and social-related treatment recommendations made by the CGA staff. Another striking finding was that 36% were very satisfied with their relationship with the CGA and with their ability to discuss things with the CGA staff when they needed to do so. Previous studies have shown that maximal involvement of primary care physicians in decision-making improves the rate of recommendation implementation [24, 39]. Furthermore, geriatric training for primary care physicians can reduce obstacles in the way of better treatment for elderly patients with complex medical problems [40]. Thus, in order to maximize the effectives of CGA there is a need for broader involvement of primary care physicians including the provision of geriatric training for them [41]. In individual cases, where the patient has complex geriatric problems and, in particular, when they also have high comorbidity rate, it is reasonable to suggest to the patient, the caregiver, and the primary care physician that the unit staff participate in the implementation of geriatric recommendations.

### Strengths and limitations

The strengths of the present study include a relatively large sample size of patients, an 8-year study period, and a rigorous computerized recording of the geriatric assessment in clearly comprehensible data entry fields. These factors enabled us to collect a broad database on the unit’s patients.

However, the present study also has some significant limitations. First, it is a retrospective study so there could be many confounders that were not taken into account. Because of the nature of the study we were not able to conduct a survey among the primary physicians whose patients participated in the study with the aim of clarifying the reasons for implementing or not implementing the recommendations. Furthermore, we did not check the actual implementation of recommendations by the patients, but only the initiation of the process when the primary physician changed the patient’s medications or gave any type of referral. Yet, it has been shown previously that there is an association between the implementation of recommendation by the primary physician and adherence to the intervention plan by the patient [42]. A previous study found that recommendations that were defined by the geriatrician as “very important” had a higher rate of implementation by the primary care physician [15]. Although the geriatrician emphasized the important recommendations in their discussion with the patient and caregiver at the end of the assessment, they were not emphasized in the letter from the geriatrician to the patient’s physician. We view this as one of the limitations of the intervention.

There are several additional limitations that are associated with the retrospective nature of the study. We were not able to evaluate associations between the rate of implementation of recommendations with new vs. old diagnoses and with acute vs. chronic changes in the patients’ status. Although the majority of patients came to the unit accompanied by caregivers this information was not available at the time of the study so we could also not assess the effect of the absence of caregivers on the rate of implementation of recommendations.

In addition, because this was a retrospective study we could not determine the reason for non-implementation of recommendations by primary care physicians or to assess whether any other action was taken instead of the given recommendations.

In summary, in the present study only multiple comorbid conditions, but no other patient or doctor characteristics, was associated with the low rate of implementation of geriatric recommendations by primary physicians.

## Abbreviations

BI: Barthel index
CCI: Charlson comorbidity index
CCITS: Charlson comorbidity index total score
CDT: Clock drawing test
GDS-15: 15-item geriatric depression scale
GEMU: Geriatric evaluation and management unit
High DS: High depression score
MMSE: Mini-mental state examination
MoCA: Montreal cognitive assessment
OARS-IADL: Older Americans resources and service instrumental activity of daily living
OCGA: The outpatient comprehensive geriatric assessment
OCGAU: OCGA unit
PHQ-9: 9 item patient health questionnaire.
